# Investigation of Effect of the Colistin Loading Dosage on the clinical, Microbiological, and Laboratory Results in *Acinetobacter baumannii* Ventilator-Associated Pneumonia /Pneumonia

**DOI:** 10.1155/2022/5437850

**Published:** 2022-08-28

**Authors:** Ayşegül Seremet Keski̇n, Derya Seyman, Kübra Demir Önder, Filiz Kizilateş, Olgun Keski̇n

**Affiliations:** ^1^University of Health Sciences Antalya Training and Research Hospital, Infectious Disease and Clinical Microbiology, Antalya, Turkey; ^2^University of Health Sciences Antalya Training and Research Hospital, Department of Pulmomology., Antalya, Turkey

## Abstract

**Materials and Methods:**

Adult patients administered colistin with and without LD for MDR *Acinetobacter baumannii* VAP/pneumonia in intensive care units (ICUs) in a tertiary teaching hospital between 1 January 2018 and 31 December 2019 were included in this retrospective cohort study. The primary endpoint was an assessment of clinical and microbiological success between treatment groups. Secondary endpoints included 14- and 30-day mortality and development of nephrotoxicity.

**Results:**

A total of 101 patients were included (colistin with LD, *n* = 57; colistin without LD, *n* = 44). No significant difference in clinical success was observed between groups (73.7% versus 77.3%; *p*=0.670). In patients receiving colistin with LD, the microbiological success rate increased from 65.9% to 71.9%, but there was no statistically significantly difference (*p*=0.510). In terms of using combination therapies with carbapeneme and/or tigecycline, there was no significant difference between treatment groups (*p*=0.30). The rates of 14- and 30-day mortality were similar between groups. The colistin with LD group had a higher rate of nephrotoxicity compared to the other group (52.6% versus 20.5% *p*=0.001). The clinical and microbiological response times were found significantly higher in the colistin with LD group (*p*=0.001; *p*=0.017).

**Conclusion:**

Colistin with LD was associated with a higher risk of nephrotoxicity and was not related to clinical success, microbiological success, and prolonged survival. Randomized comparative studies are needed to confirm the efficacy of LD colistin regimen on MDR *Acinetobacter* infection.

## 1. Introduction

The concerns about the emergence of MDR microorganisms, especially in clinically significant carbapenemase-producing *Klebsiella pneumoniae, Pseudomonas aeruginosa,* and *Acinetobacter baumannii*, continue to increase worldwide. The treatment options for MDR Gram-negative bacteria (GNB) infection are limited to several antibiotics such as colistin, tigecycline, aminoglycosides, and trimethoprim-sulfamethoxazole, due to the lack of newly developed antimicrobial molecules. The main therapy option for MDR GNB infections is colistin. Colistin has been reused over the past two decades for MDR GNB infection, and it is still the cornerstone of monotherapy and combination therapy as the salvage therapeutic option. Colistin has in vitro activity against MDR GNB, including carbapenem resistance *A.baumannii* (CRAB), but there is different evidence regarding the clinical and microbiology efficacy of colistin in real-life studies. The reasons for these discrepancies include different infection types, the pathogen caused by infection, the resistance profile of the pathogen, and the severity of the underlying illness. Moreover, the different commercial forms and dose strategies of colistimethate sodium can be responsible for these conflicting results. Therefore, the optimal dosing strategy of colistin is still uncertain. The critical unanswered question related to colistin is the impact of LD. It is thought that the use of LD may reduce the emergence of resistance and increase the rates of clinical success and microbiological success. Today, the administration of LD is supported in severe and life-threatening MDR GNB infections [1].

Colistin is a nephrotoxic drug; however, the raised MDR GNB infection rates have triggered an increase in colistin usage. It is still the most crucial antimicrobial agent since the beginning of the 2000s. Recent pharmacokinetic/pharmacodynamic (PK/PD) studies propose an LD and a high-dose maintenance treatment to reach adequate colistin concentrations [2]. Nevertheless, data on whether colistin LD regimens increase the risk of renal toxicity and better clinical and microbiology efficacy are rare [3, 4].

This study aimed at investigating the clinical success, microbiological success, and colistin-related nephrotoxicity between patients receiving colistin with and without LD to treat ventilator-associated pneumonia (VAP)/pneumonia caused by MDR *A. baumannii.*

## 2. Materials and Methods

### 2.1. Study Design and Data Collection

This was a retrospective cohort study on the microbiological success, clinical effect, and colistin-related nephrotoxicity in patients treated with intravenous (IV) colistin with and without LD for MDR *A. baumannii* VAP/pneumonia. The study was performed between 1 January 2018 and 31 December 2019, in a tertiary care academic and community-based hospital in Antalya, Turkey, with a total of 904 beds with 87 intensive care beds. This study was approved by the institutional review board of Antalya Education and Research Hospital ethics committee (19.04.2018-8/7).

The intensive care patient assessment forms, including the data for demographic characteristics, underlying diseases, diagnosis on admission, clinical findings and symptoms, invasive procedures, infection site, disease severity scores, length of hospital stay, culture results as well as microbiological success time, clinical success time, and treatment regimens and mortality, were completed daily by infectious diseases specialists. Patients diagnosed with MDR *A. baumannii* VAP/pneumonia, receiving colistin for the first time for at least 72 hours, were identified according to clinical criteria using intensive care patient assessment forms.

The use of combination therapy, defined as having two or more antibiotics, was noted regardless of in vitro activity. The extent of comorbid illnesses was evaluated by using the Charlson comorbidity index. Sequential Organ Failure Assessment (SOFA) and Acute Physiology and Chronic Health Evaluation (APACHE II) scores were assessed at diagnosis time of MDR *A. baumannii* VAP or pneumonia.

In Turkey, the commercially available form of colistimethate sodium (Colimycin; Kocak Farma, Istanbul, Turkey) was Colimycin during the study period, and each vial contained 150 mg of colistin base activity. The LD group was defined if patients received a loading dose of 300-mg colistin infusion followed by a maintenance dose of 150 mg every 12 hours. The control group received colistin 150 mg every 12 hours. In addition, all of the patients received 75 mg of inhaled (IH) colistin close to the time of IV colistin therapy twice daily. The 2-drug or 3-drug combination therapy, including carbapenems, tigecycline, and others (trimethoprim-sulfamethoxazole, fosfomycin), was used concurrently with IV and IH colistin.

### 2.2. Study Population

Adult patients diagnosed with VAP and pneumonia, according to diagnosis criteria published by the National Healthcare Safety Network, were included in this study [5]. Patients younger than 18 years of age with a high baseline creatinine level and co-infection or bacteremia were excluded.

### 2.3. Microbiological Methods

Respiratory samples were collected into a sterile container and transported immediately to Clinical Microbiology Department. The samples were plated on blood agar and eosin methylene blue agar. Plates were incubated aerobically overnight at 37°C. Matrix-assisted laser desorption ionization-time of flight mass spectrometry (VITEK MS, BioMerieux, France) was used to identify bacteria. Antibiotic sensitivity tests were performed by the VITEK2 (BioMerieux, France) system according to EUCAST criteria. MDR *A. baumannii* was defined as the presence of resistance to at least one antimicrobial drug in three or more antimicrobial categories.

### 2.4. Efficacy Endpoints and Definitions

The primary endpoints were clinical and microbiological successes. Clinical success was defined as the recovery of symptoms and signs of infection and improvement of relevant laboratory results after the treatment. The therapeutic failure was determined whether VAP/pneumonia-related clinical findings worsened or persisted without improvement after four days of treatment. The microbiological success was defined as a negative culture for *A. baumannii* 2–5 days after initiating the treatment at the earliest and in the subsequent cultures until the end of treatment, and if not provided, this was determined as a microbiological failure. During colistin administration, other secondary endpoints were 14-day and 30-day all-cause mortality and nephrotoxicity. These definitions were performed from notes in intensive care patient assessment forms. The nephrotoxicity was defined according to RIFLE (Risk, Injury, Failure, Loss of kidney function, and End-stage kidney disease) criteria (D). RIFLE criteria include the increase of serum creatinine of at least 50% from baseline (defined as Risk), doubled serum creatinine level from the baseline (defined as “Injury”), or three times increase in serum creatinine (defined as “Failure”). Nephrotoxicity was evaluated on a daily basis.

### 2.5. Statistical Analysis

Statistical analysis was performed using IBM SPSS Statistics for Windows, version 23.0 (IBM Corp. Armonk, NY). The normality assumptions were controlled by the Shapiro–Wilk test. Descriptive analyses were presented using median (0.25–0.75 percentiles) or *n* (%) values, where appropriate. Categorical data were analyzed by Pearson chi-square and Fisher's exact test. The Mann–Whitney *U* test analyzed the non-normally distributed numerical data. The Kruskal–Wallis test compared nonparametric variables between groups, and the Bonferroni–Dunn test was used as a posthoc test for significant cases. Multivariate logistic regression analysis determined independent risk factors associated with mortality. The variables with a significant association with mortality in the univariate analyses were further tested in the multivariate model. Since the Charlson comorbidity index, APACHE, and SOFA scores are highly correlated, a separate regression model was created for each variable. A *p* value of less than 0.05 was considered statistically significant.

## 3. Results

### 3.1. Patients and Clinical Characteristics

During the study period, a total of 101 patients met the study criteria, as 57 patients were treated with colistin with LD and 44 patients with colistin without LD. The median age of patients in the colistin with LD group was 63 years (46–74), and 69 (56–78) in the colistin without LD group.

Baseline characteristics of groups were generally similar in terms of demographics, ICU hospitalization reasons, comorbidities, and severity of illness (Table 1). In both groups, the most common ICU hospitalization reason was cerebrovascular diseases. Diabetes mellitus (DM) was the most common underlying disease. The patients were critically ill in both groups, with a median APACHE II score of 19 vs. 19 (*p*=0.532) and a Charlson comorbidity index of 6 vs. 6 (*p*=0.14). Furthermore, there was no difference in the SOFA score between groups (6.5 vs. 7, *p*=0.46). The length of previous ICU stay at the time of positive culture was longer in the colistin with LD group than in the group without LD; however, the difference was not statistically significant (21 vs. 14 days; *p*=0.06). The median length of total ICU stay was similar between the groups (*p*=0.407). A total of 61 patients (60.3%) had VAP. VAP was a more common infection than pneumonia in the colistin with LD group. The number of patients with VAP and pneumonia was not statistically significantly different in both groups (*p*=0.061). The mean duration of colistin therapy was similar as well (10 vs. 11 days, *p*=0.181) in both groups.

All patients had combination therapy, including carbapenem (24.6% vs. 27.3%), tigecycline (66.7% vs. 54.5%), and other drugs (8.8% vs. 18.2%), in the LD group and without LD group, respectively.

### 3.2. Clinical and Microbiological Outcomes and Nephrotoxicity

Clinical success was achieved in 73.7% of patients in the colistin with LD group and 77.3% in the colistin without LD group (*p*=0.679). The rate of microbiological success was similar between the colistin with LD and without LD groups (71.9% vs. 65.9%, respectively; *p*=0.515) (Table 2). However, interestingly, the colistin with LD group had a significantly shorter clinical success time compared to the colistin without LD group (3–5 vs. 5–7 days, *p*=0.001). Moreover, a similar relationship was determined in microbiological success time (4 vs. 6 days; *p*=0.017). 40.4% of patients who received LD and 38.6% of patients who did not receive LD had mortality (*p*=0.861). 14- and 30-day mortality rates were similar between groups (*p*=0.286; *p*=0.641). Contrary to expectation, in the colistin with LD group, the median day of mortality was statistically shorter than the colistin without LD group (6 vs. 12 days, *p*=0.032). Also, there was no significant difference in the ICU length of stay (median 36 vs. 37 days).

Nephrotoxicity was determined in 39 (38.6%) of all patients. The nephrotoxicity rates were 52.6% (30/57) in the colistin with LD group and 20.5% (9/44) in the without LD group (*p*=0.001). Although baseline creatine levels were significantly lower in the colistin with LD group (*p*=0.007), the colistin with LD group had a significantly higher nephrotoxicity rate than the colistin without LD group (*p*=0.001). When the patients were divided according to whether nephrotoxicity occurred or not, there was also no difference in clinical success (66.7% vs. 80.6%; *p*=0.113) and microbiological success (74.4% vs. 66.1%, *p*=0.383). The patients developing nephrotoxicity had significantly higher SOFA scores and 30-day mortality rates, and shorter lengths of ICU stay (*p*=0.026; *p*=0.046; *p*=0.034, respectively)

According to univariate analyses, the patients with mortality were older, had underlying diseases (DM, coronary artery diseases, and malignancy), had higher APACHE II, SOFA, and Charlson comorbidity scores, higher rate of VAP and using carbapenem, and lower rates of clinical response and bacteriological response than survived patients (Table 3). Also, the mortality rate was numerically higher in patients developing nephrotoxicity (51.3%) than in patients without nephrotoxicity (32.3%). However, nephrotoxicity was not determined as a risk factor for mortality (*p*=0.057).

There was no significant difference in clinical success, microbiological success, clinical success time, nephrotoxicity, mortality, and mortality day between the three combination therapy groups (Table 4). In the colistin with LD group, the mean bacteriological success time of patients receiving carbapenem was shorter than that of patients receiving tigecycline and other drugs (3 vs. 5 and 6; *p*=0.010).

## 4. Discussion

VAP/pneumonia caused by MDR GNB, especially CRAB, is one of the most difficult-to-treat among intensive care unit (ICU)-acquired infections. A better understanding of the management of these infections is necessary. Unfortunately, treatment options have been limited. In the last two decades, colistin, used either alone or in combination with carbapenems, aminoglycosides, rifampin, fosfomycin, trimethoprim-sulfamethoxazole, or tigecycline, has been an effective treatment of life-threatening infections caused by MDR GNB. Concerning its effectiveness, some studies have revealed that the LD colistin regimen not only increases clinical and bacteriological outcomes but may not also increase nephrotoxicity.

The present study evaluated the use of colistin with LD versus colistin without LD to treat VAP/pneumonia caused by MDR *A. baumannii*. According to the results of our study, the mortality rate, and clinical and microbiology outcomes were not statistically significantly different between both groups. The clinical and microbiology success duration was better in the patients treated with LD rather than without LD. The nephrotoxicity rate was significantly higher among patients receiving LD colistin. The duration of mortality day was significantly shorter in the colistin with LD group. This condition was considered to be due to developing nephrotoxicity in more than half of deceased patients.

According to international consensus guidelines performed by various societies in 2019, the dosing strategy of colistin was revised as a loading dose of 9 million international units (IU) followed by a high maintenance dose (4.5 million IU every 12 hours) [6]. Elefritz et al. compared patients receiving high-dose (HD) colistin with an LD regimen between September 2012 and February 2014 and a standard (low dose) regimen (6 million IU of colistin per day) with no LD between April 2009 and August 2012 for MDR Gram-negative pneumonia. They did not find a statistically significant increase in clinical cure and nephrotoxicity rates, and a decrease in time to clinical cure, length of ICU and hospital stay, and mortality rate after application of the colistin with LD or HD [7]. However, several studies have reported the effectiveness of LD colistin monotherapy and/or combination therapies on MDR GNB infections [8–11]. In the literature, especially there are studies performed by Katip et al. on the efficacy and safety of LD colistin for the treatment of MDR or carbapenem-resistant *A. Baumannii*. However, the results of these studies were different from each other, and the main cause may be other infections accompanying *A. baumanii* infection (concomitant infections). The effectiveness of LD colistin on clinical response, mortality, and nephrotoxicity rates were not found in the study including 255 patients having co-infection [9]. Another study involving 383 patients without co-infection indicated that the clinical response (54.8% vs. 55.2%), the microbiological response (54% vs. 57.9%), nephrotoxicity (32.2% vs. 56.7%), and the survival rate (37.9% vs. 42%) were significantly higher in the LD group [10]. In another study performed among patients with Gram-negative bacilli infections in Tunisia, the clinical cure level in the LD group was reported to be much higher than in the standard-dose colistin group (63% vs. 41.3%) [9]. In a study by Alp et al. [12], which included 52 VAP patients administered colistin with and without LD for *A. baumanii* in Turkey, the clinical cure rate and the bacteriological clearance rate of the LD group (19%) were not statistically higher than standard group (47.6% vs. 56.7%; 80% vs. 81%, respectively). Although the patients without co-infection were included in our study, LD colistin was not determined to be effective on clinical and microbiological responses. On the other hand, the clinical and microbiology success duration was significantly shorter associated with LD than without LD.

The first meta-analysis, including randomized controlled trials and observational studies, evaluated the efficacy and safety of colistin LD. According to this meta-analysis, colistin LD was associated with a significantly higher microbiological response, but there was no significant difference in clinical cure, mortality, and nephrotoxicity in patients receiving colistin LD [4].

The tissue concentrations of colistin are different. The lung parenchyma penetration following IV administration is not well. However, nebulized administration of colistin achieves high lung tissue concentrations (at the infection site) with minimal systemic toxicity [13]. Choe et al. investigated the effectiveness of three different methods of colistin treatment (colistin with LD, colistin without LD, colistin with LD, and adjunctive aerosolized colistin) in critically ill patients with VAP/pneumonia caused by carbapenem-resistant Gram-negative bacteria. The study demonstrated that microbiological eradication was significantly higher at the rate of 60% in patients treated with adjunctive aerosolized colistin in combination with LD (while it was 31% and 33% in the other two groups), 30-day mortality was significantly lower, and the clinical response was better than other two groups without an increase in nephrotoxicity [14]. In agreement with these findings, all of our patients were administered aerosolized colistin together with IV colistin to evaluate the efficacy of the LD.

Colistin is usually used together with rifampin, fosfomycin, carbapenem, tigecycline, and trimethoprim-sulfamethoxazole as a combination therapy for the management of MDR-AB infections. The efficacy of colistin monotherapy or combination therapy on carbapenem-resistant AB infections is still controversial [15]. Recently, the studies have not demonstrated the superiority of combination therapy to monotherapy despite the synergistic effect between colistin and meropenem in vitro against carbapenem-resistant GNB [16, 17]. Similarly, Katip et al. compared LD colistin mono- and combination therapy with meropenem against carbapenem-resistant *A. baumannii* infections, indicating that LD of colistin combined with meropenem was better approximately 1.2 times more than LD colistin monotherapy regarding clinical and microbiological responses. However, these results were not statistically significant [18].

In another study, the same authors compared the same therapy groups in critically ill patients with CRAB infections from 2015 to 2017 according to the propensity score matching analysis results using the logistic regression model. Colistin plus meropenem combination therapy was associated with a significantly lower risk of 30-day mortality, and higher clinical and microbiological responses, and did not increase nephrotoxicity compared to colistin monotherapy [19]. In our center, colistin is used in combination with a higher dose of meropenem (2 gr prolonged infusion three times per day), tigecycline, and trimetoprim sulfamethoxazole as combination therapy, and the colistin monotherapy is not used for carbapenem-resistant *A. baumannii* infections. In this study, all patients received combination therapy with colistin with/without LD, and we did not find any effect of LD on clinical and bacteriological success. Moreover, there was no significant efficacy on clinical response, microbiological response, and overall mortality when cancer patients had LD colistin to treat extensively drug-resistant *Acinetobacter baumannii*; however, the nephrotoxicity rate was significantly higher in the LD group [20]. LD colistin was found to be associated with increased nephrotoxicity in our study.

Nephrotoxicity is the most feared critical adverse effect of colistin, especially with the newly recommended LD regimen. However, published data regarding the safety (nephrotoxicity rate) of colistin LD differ, such as data on improving clinical and bacteriological outcomes, ranging from 6% to 56–58% [21]. A meta-analysis of five randomized controlled trials (RCTs), including four studies with administration of LD, reported that the incidence of nephrotoxicity was 36.2% [22]. Previous studies did not find a higher risk of nephrotoxicity despite increasing daily doses of colistin from 6 MIU to 9 MIU additional LD [7, 11]. In contrast, a prospective cohort study performed between the periods 2006–2009 and 2012–2015 reported that the currently recommended colistin LD and HD maintenance regimen was associated with an increased nephrotoxicity rate [23]. Choe et al. did not demonstrate a difference in nephrotoxicity rates between the colistin with LD and adjunctive aerosolized colistin group, the colistin with LD group, and the colistin without LD group [14]. In our study, the rate of nephrotoxicity was significantly more frequent in the colistin with LD group (52.6% vs. 20.5%). Similarly, in a study performed among patients receiving LD colistin for carbapenem-resistant *A. baumannii* infections by Katip et al., the nephrotoxicity rate was reported to be close to (56.7%) that of our study [10]. In Turkey, Alp et al. reported that the nephrotoxicity rate was higher at the rate of 50% in patients receiving colistin with LD regimen than colistin without LD regimen (27.3%) for MDR *A. baumannii* VAP. However, the difference was not statistically significant (*p*=0.099), and the mortality rate increased from 35.5% to 76.2% in patients with nephrotoxicity (*p*=0.004) [12]. In our study, no significant difference in mortality rate was determined in patients with nephrotoxicity.

## 5. Conclusion

The results of our study do not support higher efficacy and safety of LD, and the patients receiving LD have a greater likelihood of experiencing nephrotoxicity. Therefore, randomized controlled trials instead of observational studies are needed to explain whether the colistin LD regimen is necessary for critically ill patients. In routine practice, the increased rate of nephrotoxicity in these patients should be considered while deciding whether to administer LD by clinicians.

### 5.1. Limitations

The limitations of this study are the lack of evaluation of confounding factors (e.g., nonsteroidal anti-inflammatory drugs, ultrafiltration, radiocontrast agent, diuretic agents) that may cause nephrotoxicity other than colistin.

## Figures and Tables

**Table 1:** Demographic and clinical data of patients.

Variables	Colistin with LD	Colistin without LD	*p*
	(*n*/%)	(*n*/%)	
Age in years (min-max)	63 (46–74)	69 (56–78)	0.149
Male gender	37 (64.9)	31 (70.5)	0.556

Reason for intensive care unit admission			
Cerebrovasculer diseases	17 (29.8)	15 (34.1)	
Infectious diseases	11 ((19,3)	9 (20.5)	
Postoperative care	11 (19.3)	3 (6.8)	
Cardiovascular diseases	3 (5.3)	2 (4,5)	
Trauma	9 (15.8)	4 (9.1)	0.202
Pulmonary diseases	6 (10.5)	11 (25)	

Underlying disease			
Diabetes mellitus	24 (42,1)	18 (40.9)	0.904
Chronic obstructive lung disease	12 (21.1)	13 (29.5)	0.327
Chronic cardiac disease	21 (36.8)	11 (25)	0.205
Malignancy	11 (19.3)	6 (13.6)	0.451
Charlson comorbidity index	6 (3–8)	6 (5–8)	0.140
APACHE score	19 (17–22)	19 (18–22)	0.532
SOFA score	7 (5–9)	6.5 (5–9)	0.465
The length of previous intensive care unit stay	21 (11–31)	14 (7–23)	0.060
Total intensive care unit stay	37 (25–67)	36 (21–58)	0.407

Antibiotic used in combination			
Carbapenem	14 (24.6)	12 (27.3)	
Tigecycline	38 (66.7)	24 (54.5)	0.305
Other antibiotics	5 (8.8)	8 (18.2)	

Pnemonia/VAP			
Pneumonia	18 (31.6)	22 (50)	0.061
VAP	39 (68.4)	22 (50)	

APACHE: Acute Physiology and Chronic Health Evaluation; SOFA: Sequential Organ Failure Assessment; VAP: ventilator-associated pneumonia.

**Table 2:** Clinical and microbiological success evolution of patients.

	Colistin with LD	Colistin without LD	*p*
	(*n*/%)	(*n*/%)	
Clinical success	42 (73.7)	34 (77.3)	0.679
Microbiological success	41 (71.9)	29 (65.9)	0.515
Clinical success time	5 (3–5)	5 (5–7)	**0.001**
Microbiological success time	4 (3–6)	6 (4–7)	**0.017**
Mortality	23 (40.4)	17 (38.6)	0.861
Mortality day	6 (4–19)	12 (8–21)	**0.032**
Basaline creatine	0.6 (0.5–0.7)	0.8 (0.6–0.9)	**0.007**
Nephrotoxicity	30 (52.6)	17 (38.6)	**0.001**

**Table 3:** Comparison of patients' characteristics according to mortality.

Variables	Mortality		*p*
	No (*n* = 61)	Yes (*n* = 40)	
Age in years (min-max)	62 (43–74)	70 (56–78)	**0.032**
Male gender	41 (67.2)	27 (67.5)	0.976

Underlying disease			
Diabetes mellitus	20 (32.8)	22 (55)	**0.027**
Chronic obstructive lung disease	12 (19.7)	13 (32.25)	0.144
Chronic cardiac disease	14 (23)	18 (45)	**0.020**
Malignancy	6 (9.8)	11 (27.5)	**0.020**
Charlson comorbidity index	5 (3–6)	8 (6–9)	**<0.001**
APACHE score	19 (17–20)	21.5 (18–22)	**<0.001**
SOFA score	6 (5–8)	9 (7–10)	**<0.001**
The length of previous intensive care unit stay	19 (10–31)	15 (9–23)	0.242
Total intensive care unit stay	44 (29–71)	32 (18–57)	0.006

Antibiotic used in combination			
Carbapenem	14 (23)^a^	12 (30)^a^	
Tigecycline	35 (57.4)^a^	27 (67.5)^a^	**0.041**
Other antibiotics	12 (19.7)^a^	1 (2.5)^b^	

Pneumonia/VAP			
Pneumonia	30 (49.2)	10 (25)	
VAP	31 (50.8)	30 (75)	**0.015**

APACHE: Acute Physiology and Chronic Health Evaluation; SOFA: Sequential Organ Failure Assessment; VAP: ventilator-associated pneumonia. Data are presented as median (IQR) and *n* (%). Mann–Whitney U test, Pearson chi-square test, Fisher's exact test.

**Table 4:** Comparison of study outputs according to antibiotic used in combination

Variables	Colistin							
	Without LD			*p*	With LD			*p*
	Carbapenem (*n* = 12)	Tigecycline (*n* = 24)	Other (*n* = 8)		Carbapenem (*n* = 14)	Tigecycline (*n* = 38)	Other (*n* = 5)	
Clinical success	9 (75)	18 (75)	7 (87.5)	0.794	9 (64.3)	28 (73.7)	5 (100)	NA
Microbiological success	9 (75)	14 (58.3)	6 (75)	0.573	7 (50)	29 (76.3)	5 (100)	NA
Clinical success time	5 (4–6)	6 (5–7)	5 (5–7)	0.168	3 (3–4)	5 (4–5)	5 (4–7)	0.076
Microbiological success time	7 (5–7)	6.5 (4–8)	4 (4-5)	0.185	3 (3–3.5)	5 (3–6)	6 (6–7)	0.010
Nephrotoxicity	3 (25)	4 (16.7)	2 (25)	NA	5 (35.7)	23 (60.5)	2 (40)	0.264
Mortality	5 (41.7)	11 (45.8)	1 (12.5)	NA	7 (50)	16 (42.1)	0 (0)	0.157
Mortality day	12 (8–20)	18 (8–30)	10 (10)	0.748	4 (4-8)	6.5 (4.–23.5)	-	0.211

Data are presented as median (IQR) and *n* (%). Kruskal–Wallis test, Mann–Whitney U test, Pearson chi-square test. NA.

## Data Availability

The authors can also make data available on request through a data access committee, institutional review board, or the authors themselves.
